# Bayesian Integration of Bronchoalveolar Lavage miRNAs and KL-6 in Progressive Pulmonary Fibrosis Diagnosis

**DOI:** 10.3390/diagnostics15101257

**Published:** 2025-05-15

**Authors:** Piera Soccio, Valerio Longo, Corrado Mencar, Pasquale Tondo, Fabiola Murgolo, Giulia Scioscia, Donato Lacedonia

**Affiliations:** 1 Department of Medical and Surgical Sciences, University of Foggia, Viale Luigi Pinto, 1, 71122 Foggia, Italy; pasquale.tondo@unifg.it (P.T.); fabiola.murgolo@libero.it (F.M.); giulia.scioscia@unifg.it (G.S.); donato.lacedonia@unifg.it (D.L.); 2 Department of Computer Science, University of Bari Aldo Moro, 70125 Bari, Italy; v.longo20@studenti.uniba.it (V.L.); corrado.mencar@uniba.it (C.M.); 3 Institute of Respiratory Diseases, Policlinico Riuniti of Foggia, 71122 Foggia, Italy

**Keywords:** progressive pulmonary fibrosis, genetic biomarkers, Bayesian model

## Abstract

**Background/Objectives**: Progressive pulmonary fibrosis (PPF) represents one of the most severe and complex challenges in respiratory medicine, characterized by a rapid decline in lung function and often poor prognosis, making it a priority in research on interstitial lung diseases (ILDs). The aim of this study is to correlate classical clinical features and three genetic biomarkers with the diagnosis and prognosis of progressive pulmonary fibrosis in ILDs. **Methods**: This study involved 19 patients with progressive pulmonary fibrosis (PPF) and 20 patients with non-progressive pulmonary fibrosis (nPPF) from the S.C. of Respiratory System Diseases at the Policlinico of Foggia (Italy) between 2015 and 2022. All participants underwent pulmonary function tests (PFTs), a 6 min walk test (6MWT), and bronchoalveolar lavage (BAL) sampling, following the acquisition of written consent for these procedures. Bayesian analysis with generalized linear models has been applied for both diagnostic and prognostic classification. **Results**: The proposed Bayesian model enables the estimation of the contribution of each considered feature, and the quantification of the uncertainty that is consequential to the small size of the dataset. The analysis of miRNAs such as miR-21 and miR-92a, alongside the protein biomarker KL-6, was identified as a significant indicator for PPF diagnosis, enhancing both the sensitivity and specificity of predictions. **Conclusions**: The identification of specific genetic markers such as microRNAs and their integration with traditional clinical characteristics can significantly enhance the management of patients with the disease. This multidimensional approach, which integrates clinical data with omics data, could enable more precise identification and monitoring of the disease and potentially optimize future treatments through larger studies and extended follow-ups.

## 1. Introduction

Pulmonary fibrosis is a chronic disease that results from the excessive accumulation of fibrotic tissue in the lungs, causing thickening and stiffness that compromise lung function and lead to symptoms such as dyspnea and reduced breathing capacity [1,2].

There are two primary forms of lung fibrosis: progressive and non-progressive. Progressive pulmonary fibrosis (PPF) is characterized by a continuous and irreversible decline in lung function despite treatment. Disease progression is defined by a decline in forced vital capacity (FVC), worsening symptoms, and an increased need for oxygen therapy [3]. Idiopathic pulmonary fibrosis (IPF) is one of the most severe and well-known forms of PPF, but progressive fibrosis can also occur in other interstitial lung diseases (ILDs). IPF is a chronic fibrotic lung disease of unknown origin that predominantly affects middle-aged and elderly adults. It is characterized by progressive fibrosis of lung tissue, leading to irreversible deterioration of lung function. While the clinical course of IPF is variable, it is generally associated with a poor prognosis, with a median survival ranging from 3 to 5 years from diagnosis [3]. Key symptoms include dyspnea (shortness of breath), dry and persistent cough, fatigue, and weight loss.

In addition to IPF, several ILDs can develop a progression that is characterized by irreversible fibrosis and impaired respiratory function. These include non-specific interstitial pneumonia (NSIP), which can exhibit a progressive fibrotic course, although it typically presents a more uniform fibrotic pattern compared to IPF. NSIP may be idiopathic or secondary to autoimmune diseases and can have a more favorable prognosis than other forms of progressive pulmonary fibrosis [4]. Chronic hypersensitivity pneumonitis, caused by prolonged environmental exposure, can evolve into progressive fibrosis with a clinical presentation similar to that of IPF [3]. Connective tissue disease associated with interstitial lung disease (CT-ILD), which may occur in patients with autoimmune diseases, shows an unpredictable progression and a significant effect on prognosis [5]. Fibrotic pneumoconiosis, such as silicosis and asbestosis, caused by chronic inhalation of toxic mineral dusts, results in progressive pulmonary fibrosis and impaired respiratory function [6]. Finally, fibrotic sarcoidosis, a late-stage complication of sarcoidosis, is characterized by disease-typical granulomas progressing to diffuse fibrosis, with effects on lung function similar to those found in other fibrotic ILDs [7].

The diagnosis of progressive fibrotic ILDs is based on a combination of clinical history, imaging (particularly high-resolution computed tomography, HRCT), lung function tests, and, in some cases, lung biopsy. Identifying fibrotic progression early is crucial for enhancing treatment and the clinical management of patients.

In contrast, non-progressive pulmonary fibrosis (nPPF) is characterized by the stabilization of fibrotic tissue in the lungs, without further deterioration over time. In this form, the scarring process may stop or slow significantly, allowing the patient to maintain relatively stable lung function over time. Although this condition can also occur in IPF, it is more common in other forms of pulmonary fibrosis [3].

Despite advances in medical research, there are no specific laboratory tests for the early detection of progressive fibrosis. The lack of diagnostic tools hinders the ability to accurately predict the activity of the disease and its future course. A better understanding of the underlying molecular mechanisms is therefore crucial for the development of targeted treatments and improved patient outcomes. Krebs von den Lungen 6 (KL-6) is a mucus-forming glycoprotein with a high molecular weight that is expressed on the surface membrane of alveolar epithelial cells (AEC-II) and bronchiolar epithelial cells. Recent studies have shown that elevated serum levels of KL-6 act as a prognostic indicator, correlating with decreased survival rates [8,9]. Additionally, KL-6 has been identified as a predictor of exacerbations in IPF [10], and it shows promise as a biomarker for monitoring the response to antifibrotic therapy [11]. Furthermore, KL-6 has been identified as a promising biomarker to assess the severity of fibrotic processes and disease progression in ILD, with significantly higher levels observed in patients with PPF compared to those with nPPF. The significant difference in KL-6 levels between serum and bronchoalveolar lavage fluid (BAL) highlights its potential diagnostic and prognostic value, providing information on both local and systemic aspects of ILD [12].

MicroRNAs (miRNAs), a class of small noncoding RNAs that play a key role in the post-transcriptional regulation of gene expression, also play a fundamental role in pulmonary fibrosis by modulating gene expression involved in key pathological processes such as epithelial–mesenchymal transition (EMT) and extracellular matrix deposition [13,14]. Dysfunctions in these miRNAs can alter the balance of the inflammatory response and collagen production, contributing to the development and progression of pulmonary fibrosis [15].

MiRNAs can be transported by nanometric extracellular vesicles known as exosomes. Exosomes are essential for intercellular communication and can influence physiological and pathological processes. Many molecular components of exosomes have also been associated with various diseases, suggesting a role as diagnostic, prognostic, and therapeutic monitoring tools [16,17,18]. Recent studies on exosomal miRNAs, derived from both the serum and BAL of patients with IPF, highlight their role in the pathogenesis of the disease and their potential as progression biomarkers and as targets for the development of new drugs [19,20].

The combination of clinical and genetic biomarkers, such as KL-6, miR-21, and miR-92a, represents a promising opportunity to improve early diagnosis, risk stratification, and therapeutic decision-making in patients with fibrotic ILDs. Despite advancements in imaging diagnostics and functional assessment, the early identification of fibrotic progression remains a clinical challenge. In this context, the combination of clinical and genetic biomarkers, such as KL-6, miR-21, and miR-92a, represents a promising opportunity to enhance early diagnosis, risk stratification, and therapeutic decision-making in patients with fibrotic ILDs. KL-6 reflects the damage and regenerative processes of the alveolar epithelium [8,9,10,11], while miR-21 is known for its pro-fibrotic role, as it promotes epithelial–mesenchymal transition (EMT) and extracellular matrix deposition [13,14,15]. In contrast, miR-92a plays an anti-fibrotic role, modulating angiogenesis and matrix remodeling [19,20]. The interaction between miR-21 and miR-92a may play a key role in balancing the pro- and anti-fibrotic processes, furthering our understanding of disease progression and refining the potential diagnostic and therapeutic approaches. Integrating these biomarkers into a single analytical model could improve diagnostic accuracy and allow for a more precise characterization of fibrotic progression.

In the diagnosis of pulmonary fibrosis, sample sizes vary depending on the research approach. Studies using CT analysis for drug trials have used hundreds of cohorts to validate predictive measures of disease severity [21]. On the contrary, deep learning detection methods have employed datasets with thousands of samples to ensure the accuracy of automated classification systems [22].

Meanwhile, exploratory studies on PET/CT imaging have operated with smaller patient groups to evaluate new imaging modalities [23]. These variations in sample sizes reflect the diverse methodologies and objectives in pulmonary fibrosis diagnostic research.

Obviously, the cost of a study is proportional to the sample size of the population involved. This problem is further exacerbated when studies involve complex procedures such as biomarker measurements. For example, in a recent meta-analysis of the prognostic role of KL-6 measurement in IPF, the reported studies involved 56 to 267 cases, with the latter obtained from multiple centers [24].

It is well known that small sample sizes can limit the significance of statistical studies [25]. Bayesian analysis offers a robust solution for addressing challenges posed by small sample sizes in medical studies [26,27,28,29].

It allows for the incorporation of prior information, enhancing the reliability and interpretability of results. By utilizing Bayesian methods, researchers can effectively quantify the uncertainty associated with results from small datasets, helping to make decisions for future research directions. Moreover, Bayesian approaches facilitate the combination of all available information throughout the clinical trial process, from design to analysis. This is particularly beneficial in studies of rare diseases where conventional power requirements are challenging to meet due to inherently small sample sizes and heterogeneity. This comprehensive framework enables researchers to make informed decisions, improve study designs, and improve the accuracy of treatment effect assessments in medical research.

There are several studies that have used Bayesian analysis for the diagnosis of pulmonary fibrosis. Meltzer et al. [30,31] developed a model to diagnose IPF by applying Bayesian probit regression modeling to gene expression profiles of lung tissue. Cottin et al. [32] identified factors that influence the clinical likelihood of a diagnosis of IPF and used a Bayesian framework to integrate radiological and histopathological features to estimate the post-test probability of diagnosis. Another study also developed a model and app using Bayesian additive regression tree to assist radiologists in diagnosing the pulmonary fibrosis pattern [33]. Additionally, Bayesian network analysis has been used to integrate the extracellular vesicle proteome and clinical information for the diagnosis of IPF [34]. Other studies use Bayesian methods to evaluate drug therapies for the treatment of IPF [35,36,37,38].

Given the limited sample size (39 patients), this study should be considered exploratory in nature. While Bayesian methods are particularly suitable for modeling with small datasets, we acknowledge the potential for overfitting even within this framework. To mitigate this risk, we employed a model selection strategy based on the Expected Log Predictive Density using Leave-One-Out Cross-Validation (ELPD-LOO), which helps assess the generalization performance of the models. Nonetheless, the findings require validation in larger, independent cohorts before clinical translation.

To the best of our knowledge, while research into pulmonary fibrosis has increasingly focused on the role of miRNAs as potential biomarkers and therapeutic targets, the application of Bayesian analysis specifically to miRNA biomarkers in this context appears to be limited. In this paper, we present the results of a Bayesian analysis conducted on a small cohort of 39 patients with lung diseases. The aim of this study was to investigate the relationship between classical clinical parameters and three genetic biomarkers in the diagnosis and prognosis of progressive pulmonary fibrosis.

## 2. Materials and Methods

### 2.1. Population

This study involved 19 patients with PPF and 20 patients with nPPF from the S.C. of Respiratory System Diseases at the Policlinico of Foggia (Italy) between 2015 and 2022. The classification of patients followed the criteria established by Hambly et al. [39] to define the progression of the disease. Written informed consent was obtained from all participants, and ethical approval was granted by the Ethics Committee of Policlinico of Foggi, adhering to the principles of the Declaration of Helsinki. Ethical considerations precluded the inclusion of healthy donors. Diagnoses of various diffuse interstitial lung diseases (ILDs) were made according to the ATS/ERS/JRS/ALT guidelines [3]. The PPF group included 19 cases: 10 of idiopathic pulmonary fibrosis (IPF), 4 cases of hypersensitivity pneumonitis (HP), 2 cases of non-specific interstitial pneumonia (NSIP), and 3 cases related to rheumatologic diseases. On the contrary, the nPPF group consisted of 20 cases: 11 patients with slow-progressing IPF, 2 with HP, 3 with NSIP, and 4 with other ILDs. All participants underwent pulmonary function tests (PFT), a 6 min walk test (6MWT), and bronchoscopy with bronchoalveolar lavage (BAL) sampling, after obtaining written consent for these procedures. BAL samples from all patients were collected, aliquoted, and stored at −80 °C until analysis.

### 2.2. Bronchoalveolar Lavage: Procedure and Diagnostic Utility

Bronchoalveolar lavage (BAL) was performed for diagnostic purposes in all patients according to the guidelines of the BAL Working Group of the European Respiratory Society [40,41]. In summary, BAL is an auxiliary procedure used in fibrobronchoscopy to assess large alveolar compartments, providing both cellular and non-cellular constituents of the lower respiratory tract. During this procedure, the bronchoscope introduces 3–5 doses of 20–50 mL of saline solution into the peripheral lung, followed by the recovery of at least 30% of the volume by gentle suction to prevent damage to or collapse of the distal airways [42]. The lavage fluid was divided into aliquots, where a portion was used for studies of lung cellularity, while the rest was stored at −80 °C. The experimental procedure was carried out in accordance with the following stages [43,44,45]:

1. Exosome purification from BAL;
2. Western blotting Multiplex and Surface Marker Analysis;
3. Isolation of RNA;
4. RNA reverse transcription and expression of miRNA through q-PCR.

A detailed description of each stage is reported in the Supplementary Materials.

### 2.3. Dataset

**Figure 1:**
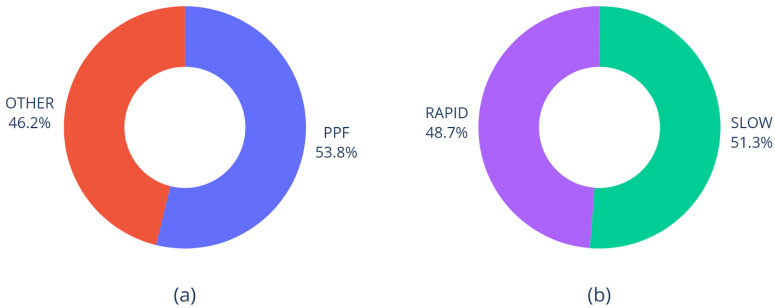
Distribution of diagnosis (**a**) and prognosis (**b**) in the dataset.

The dataset used for analysis consists of 39 samples with 11 numerical features and 4 nominal features, including 2 targets (diagnosis and prognosis), which are well-balanced (Figure 1).

Feature statistics are reported in Table 1, where 23 missing values are reported.

**Figure 2:**
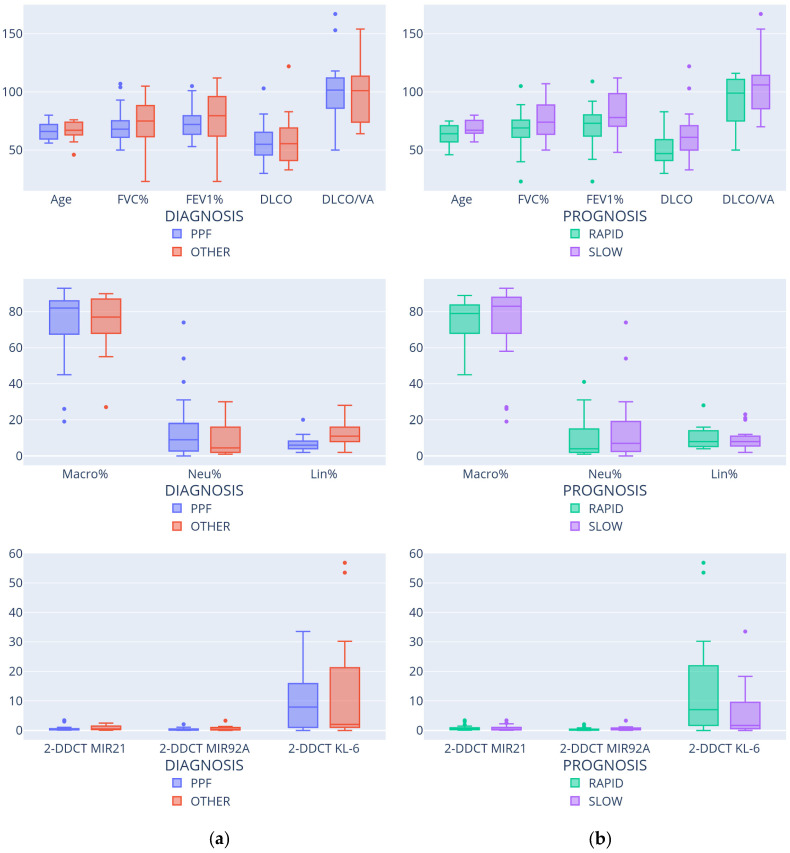
Feature distributions according to diagnosis (**a**) and prognosis (**b**).

Figure 2 reports the box plots of the numerical features conditioned to diagnosis and prognosis. 

**Figure 3:**
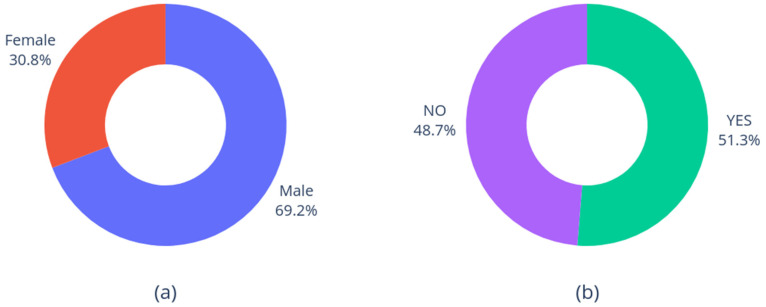
Distribution of gender (**a**) and smoking status (**b**).

Finally, Figure 3 shows the distributions of gender and smoking status. As expected, male patients are more common than females [46]; on the other hand, there is no significative difference between smokers and non-smokers.

### 2.4. Workflow

#### 2.4.1. Data Preparation

1. Missing values: no imputation methods have been considered adequate to estimate the missing values; therefore, the rows containing missing values were removed from the dataset when a processing method could not be applied with missing data.
2. Outliers: potential outliers detected from boxplots were discussed with the domain experts, and their inclusion in the dataset was eventually confirmed.
3. Normalization: we opted for the z-score normalization of all numerical features, so that each feature would be centered around the mean and would have a unitary standard deviation.

$$z=\frac{x-mean\left(x\right)}{std\left(x\right)}$$

#### 2.4.2. Feature Augmentation

Since our main interest is in understanding the possible correlation between genetic features and diagnosis or prognosis, additional features have been calculated to favor the discovery of these relationships. In particular, pairwise products have been considered to highlight the correlation among genetic features. In fact, the product of feature A and B (normalized with z-score) is positive and high if both features are high or both are low, while the product is negative if they are discordant. The pairwise product of genetic features led to the introduction of six derived features in the dataset.

#### 2.4.3. Feature Ranking and Selection

The number of features is high compared to the low cardinality of the samples. To reduce the risk of overfitting, feature selection was performed using the Wrapper method, which correlates the selected features with the selected predictive model [47]. The Wrapper method requires the iterative generation of a subset of candidate features: this is accomplished by first ranking features and then providing the selection procedure with an incremental list of features according to the rank. The following procedure has been carried out:

1. Feature ranking: the adopted method is based on three stages.
  - Random Forest feature ranking was applied [48], whereas ranking was performed by sorting the features based on their importance, which was estimated by Gini impurity.
  - Since we worked with linear models, we needed to avoid the presence of highly correlated features. To this end, a Pearson correlation matrix among features was computed, and features with an absolute correlation coefficient ≥0.5 (generally interpreted as moderate to high correlation) with higher ranked feaures were removed.
  - Finally, gender was removed because it introduced label-leakage bias in classification. Also, the DLCO/VA feature was discarded due to the high number of missing values.
2. The choice of the final feature subset was made according to the Wrapper method by using the ranked list of features obtained in the previous stage. Starting from the most important feature, several models were induced by increasingly adding new features according to the rank. These models were then evaluated according to ELPD-LOO (described henceforth), and the model showing the highest value of this measure (with the corresponding feature subset) was selected.

#### 2.4.4. Bayesian Model

Both targets (diagnosis and prognosis) are binary; therefore, the objective of the model is to estimate the probability of each class, given the selected input features. More specifically, the Bayesian model assumes a Bernoulli distribution of the target *T* (*T* being diagnosis or prognosis)

(1) $$T|x∼Bernoulli\left(p_{T}\left(x\right)\right)$$

where x is the feature vector, and the probability $p_{T}$ is defined by a generalized linear model:

(2) $$p_{T}\left(x\right)={\left(1+exp\left(\beta_{T,0}+\beta_{T}^{⊤}\cdot x\right)\right)}^{-1}$$

The proposed Bayesian model enables the estimation of the contribution of each feature considered in a transparent way. It is therefore more understandable than opaque techniques, such as deep learning models (Molnar 2022 [49]).

The Bayesian model requires the specification of the prior distributions of the linear coefficients. In this work, Student distribution has been used to specify priors because of its heavier tails than standard normal distribution.

The output of the Bayesian model is the posterior distribution of the coefficients $\beta_{0,T}$ and $\beta_{T}$ as defined by Bayes’ rule

(3) $$Pr\left(x_{os},T_{obs}\right)\propto Pr\left(\beta_{0,T},\beta_{T},x_{os}\right)\cdot PrPr\;\left(\beta_{0,T},\beta_{T}\right)\;$$

which quantifies the uncertainty of the coefficients due to the scarcity of observed data.

#### 2.4.5. Expected Log Pointwise Predictive Density (ELPD-LOO)

The generalization ability of the Bayesian model (i.e., the ability to predict the target of newly observed data) can be estimated through Leave-One Out (LOO), a general technique that estimates the target of any data point in the dataset by training a model without the data point. In the Bayesian context, the output of a model is a probability distribution, whose predictive ability is evaluated by scoring the log-probability of the outcome. The Expected Log Pointwise Predictive Density (ELPD) LOO is a scoring function suitable for evaluating the generalization ability of a Bayesian model [50].

ELPD-LOO can be used to score a Bayesian model with a specified subset of features within a feature selection process based on the Wrapper method. The model with the best EPLD-LOO will be retained as the model with the highest predictive quality.

## 3. Results

### 3.1. Feature Ranking and Selection

To keep the analysis manageable, and to focus on correlations between features of different types, we divided the features in three groups (Table 2) and considered the two highest ranked features for each group. Linear correlation among these features is negligible; therefore, all the resulting six features were retained for feature selection, according to their rank. Table 3 reports the list of features considered for each clinical problem.

For each of the two clinical problems, all the considered features were evaluated to further refine the selection. We built a Bayesian model for each individual feature and for each pair (resulting in 21 different models). In the case that the probability distribution of a coefficient gives a high probability degree to values close to zero, either for the single feature or for the pair, the corresponding feature was removed because its contribution could be irrelevant or ambiguous (i.e., in some models, the contribution of the feature to the output is positive; in others, it is negative).

Table 4 reports the list of features resulting from the workflow.

**Figure 4:**
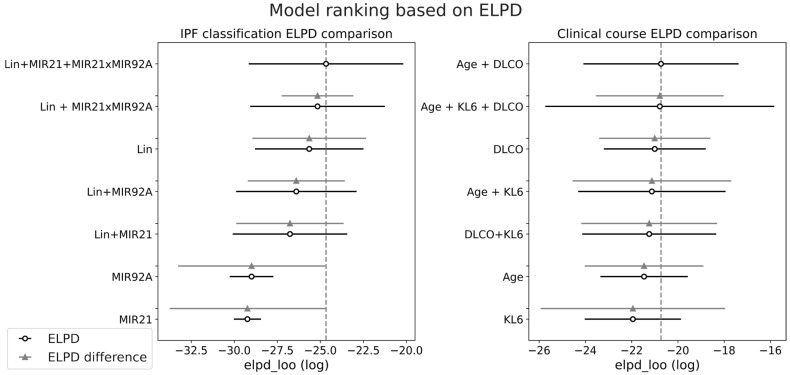
Model ranking based on ELPD computation; the circles represent the computed ELPD value, while the horizontal line is the standard error. The difference in ELPD is computed with respect to the highest ranked model.

For each subset of features in each group, we quantified the performance of the model using ELPD-LOO, which is reported in Figure 4. 

Regarding the diagnosis of IPF, the figure highlights the combination of features LIN + miR-21 + miR-21 × miR-92a as the most effective, while the exclusion of miR-21 leads to a small reduction in average ELPD. On the other hand, for prognosis estimation, the most effective models are based on AGE+DLCO, with or without KL6, which were both considered in the subsequent analysis.

### 3.2. Performance Evaluation

For each model in the ranking, and for both clinical problems, we computed the expected accuracy, sensitivity, and specificity (expected value computation is required because each model is defined by a probability distribution of the parameters). In Table 5, the performance evaluation of models for diagnosis is reported. The table shows that miRNA features, together with the LIN feature, significantly improve the expected quality of classification, in terms of accuracy, sensitivity, and specificity. In particular, the highest values of accuracy and specificity are obtained when the interaction between miR-21 and miR-92a is considered.

In Table 6, the performance results are reported for prognosis prediction. In this case, the presence of KL6 improves both accuracy and specificity when age and DLCO are also used for prediction, while sensitivity is improved when age is not considered.

In both tables, we also observe that the prediction uncertainty (as represented by the standard deviation) of the best models is small when compared with the other models, yet high in absolute terms (for example, the standard deviation of specificity of the best model for prognosis classification is 14.4% as reported in Table 6). This assessment of uncertainty is an objective indicator that more data are needed to obtain more stable results.

### 3.3. Uncertainty Evaluation

**Figure 5:**
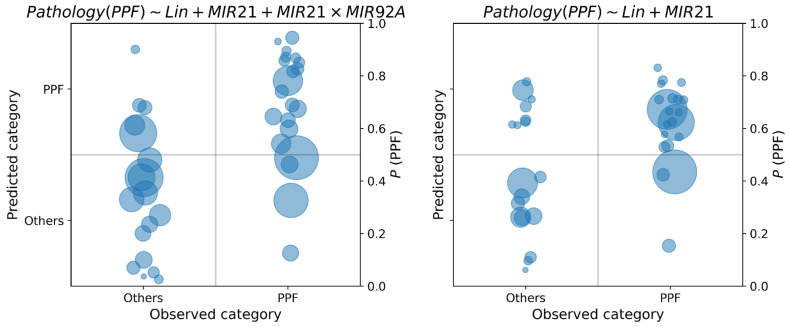
Class posterior probabilities of the best model for the diagnosis of PPF, with and without miR-21. The area of each circle is proportional to the variance of the posterior probability distribution.

**Figure 6:**
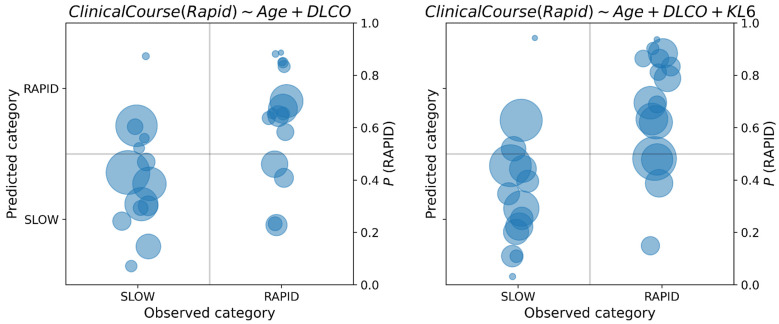
Posterior class probabilities of the best models for the prognosis of PPF, with and without KL6.

For both diagnosis and prognosis prediction, we reported the posterior probability of the class of each sample in a confusion diagram, as reported in Figure 5 and Figure 6.

In each class diagram, the x-axis shows the observed classes of PPF vs. others in diagnosis, SLOW vs. RAPID in prognosis; on the y-axis, the posterior probabilities of each data sample have been reported. As posterior probabilities are defined as probability distributions, they are synthetically represented as circles centered on the distribution mean, and radius proportional to the standard deviation. If the posterior probability is greater than 0.5, the predicted label is PPF diagnosis and RAPID for prognosis.

By recording the posterior probability of the target class, it is possible to appreciate the uncertainty in the decision: when this probability is close to the cutoff point of 0.5, classification should not be made, because the available data do not provide enough information to take a clear decision. As concerning PPF diagnosis, from Figure 5 we observe an increase in uncertainty in the classification of PPF when the compound feature miR-21 × miR-92a is not considered (right diagram). This can be observed by the concentration of the class probability distributions of PPF around the cutoff point of 0.5 when this feature is removed from the model. On the other hand, we observe a reduction in uncertainty for the opposite class when this feature is removed. Regarding the prognosis, in Figure 6 we observe an increase in uncertainty about the prediction of class probabilities when KL6 is used in the model (this is observable by the larger areas of the circles on the right diagram). However, many predictions are away from the cutoff point, thus making the classification less uncertain. We also observe the presence of predictions around the 0.5 cutoff both for the SLOW and RAPID class, which may be due to the fuzzy nature of their definition, which does not admit a sharp separation.

The presence of circles with large radii is an indication that, for some samples, the estimation of the class probability is highly uncertain. In some cases, the circles intersect the class cutoff, suggesting that no decisions should be made on these samples. On the other hand, there are samples with large radii that are far from the cutoff; in such a case, the class prediction is certain, assuming the hypothesis of the model. The reduction in the uncertainty can be achieved by increasing the number of samples. Another option to reduce the uncertainty is to use more flexible (i.e., non-linear) models, which, however, may be less interpretable than linear models, and would require validation processes (for example, k-fold cross-validation) to avoid overfitting that, in turn, requires more data.

### 3.4. Interpretation of the Linear CoefficientsUncertainty Evaluation

**Figure 7:**
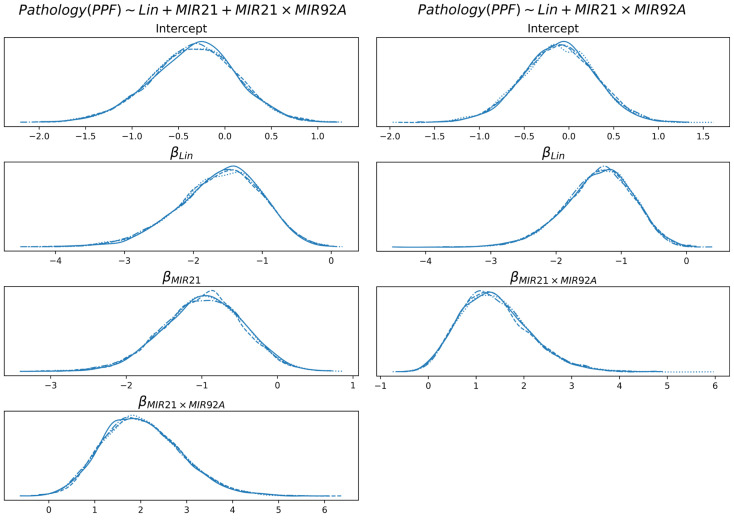
Distribution of the linear coefficients of the best model for the diagnosis of PPF, with and without miR-21. Multiple lines are reported for each distribution, which are consequences of multiple chains of Monte Carlo sampling of the distributions. The lines are highly overlapping because of sampling convergence.

In Figure 7 the linear coefficients of the logistic function (2) are reported for the diagnosis of PPF, with and without miR-21. The coefficients are described through posterior probability distributions. 

None of the coefficients shown include 0 with significant probability (intercept is not considered here). For both models and all features, the probability that a coefficient is in the interval [−0.05, +0.05] is less than 5%. This is an indication that all the coefficients concur to the definition of class probability in a coherent way. Specifically, higher values of Lin determine a decrease in probability of PPF, while the cooccurrence of miR-21 and miR-92a (i.e., either they are both low or they are both high) increases the probability of PPF. An increase in miR-21 (in the model that uses it) determines a decrease in the probability of PPF, which is partially compensated by the feature miR-21 × miR-92a.

**Figure 8:**
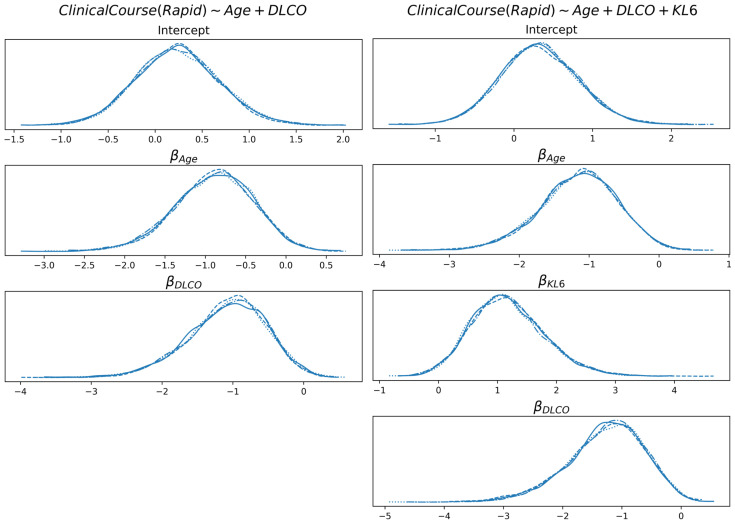
Distribution of the linear coefficients of the best model for the prognosis, with and without KL6.

In Figure 8, the linear coefficients of the logistic function are reported for the prognosis, with and without KL6. Both age and DLCO are negatively correlated with class probability (class is RAPID prognosis; therefore, the higher the age or DLCO, the higher the tendency to classify the subject with a SLOW prognosis).

On the other hand, KL6 is positively correlated (the higher the value, the higher the probability of RAPID prognosis). For both models and all features, the probability that a coefficient is in the interval [−0.05, +0.05] is less than 5.5%.

## 4. Discussion

The experimental results show that a Bayesian approach to medical diagnosis could provide interpretable information on the uncertainty of predictive models, which is particularly apparent in the case of small datasets. The Bayesian approach adopted in this study explicitly quantifies the uncertainty associated with parameter estimates, which is particularly useful in studies with limited sample sizes. Unlike traditional frequentist methods, Bayesian inference provides probability distributions for each coefficient, allowing for a more transparent assessment of each variable’s contribution to classification. This is especially relevant in exploratory studies on rare diseases or conditions with high individual variability, such as PPF.

In all cases reported in this paper, we observe bell-shaped distributions, which indicate the presence of a compact region of the parameter space where the true (yet unknown) values reside, if the modeling assumptions are correct. However, it must be noticed that data are normalized (according to z-score); therefore, the interpretation of the coefficients requires some care. As an example, the posterior distribution of the KL6 coefficient in prognosis shows that its true value is roughly between 0 and 3; this is the (uncertain) contribution of KL6 when its absolute value is around its mean plus its standard deviation.

The uncertainty in the coefficients determines the uncertainty in the classification. In fact, the confusion matrices report, for each data point, the classification that is pictorially represented by a circle whose radius is proportional to its uncertainty. It is noteworthy observing that models with a higher number of features provide more uncertain classifications than simpler models because of the propagation of uncertainty from the feature coefficients to the class.

Dealing with uncertainty makes modeling more complex, but the results are more informative and robust. For example, notwithstanding the significant uncertainty of the linear coefficients of the linear models, all of them show that the probability of being around zero or having an incoherent sign is very small: this is a robust indicator that the features are necessary for the classification. On the other hand, uncertainty quantification is essential for deciding if the available data are not enough for a robust classification and, consequently, the models are not useful for further analysis or clinical practice. In such a case, the analyst is informed that additional data are required for conclusive results.

Moreover, given the relatively small cohort analyzed in this study, the present findings should be interpreted as preliminary and hypothesis-generating. While the Bayesian framework helps mitigate some limitations associated with sample size, further validation in larger, multi-center studies is necessary to confirm these results and support their clinical applicability. It is essential to highlight that the obtained results must be validated in larger cohorts to ensure the robustness and clinical applicability of the conclusions.

From a medical viewpoint, the results provide valuable information on the disease and its potential application in clinical practice to diagnose and predict the progression of progressive pulmonary fibrosis. The findings indicate that including miRNAs such as miR-21 and miR-92a, alongside the protein biomarker KL-6, change diagnostic and prognostic accuracy for PPF. The analysis revealed that certain miRNAs are linked to the disease, indicating a potential role as diagnostic markers. However, larger studies are required to confirm their clinical applicability and define their actual predictive value.

The interaction between miR-21 and miR-92a was identified as a significant indicator for PPF diagnosis, improving both the accuracy and specificity of the predictions. This is consistent with previous studies that have highlighted the crucial role of miRNAs in modulating gene expression and the pathological processes of pulmonary fibrosis [13,51].

We demonstrated that the integration of miRNAs with protein biomarkers can provide more detailed and accurate information compared to traditional diagnostic methods. Specifically, we examine the expression of KL-6 mRNA alongside miR-21 and miR-92a. KL-6 is known for its high expression in fibrotic and interstitial lung diseases [52,53]. Analysis of KL-6 mRNA showed a significant correlation with the severity of fibrosis, confirming its role as an indicator of fibroblastic activity in the lungs. MiR-21 and miR-92a were significantly associated with PPF, suggesting that these miRNAs could play a key role in the pathogenesis of the disease. Specifically, miR-21 is known for its ability to modulate inflammatory and fibrotic responses [54,55], while miR-92a is involved in the regulation of cellular metabolism and proliferation [56,57].

Integrating miRNAs with KL-6 improved the model’s predictive capacity compared to single biomarkers, suggesting a potential benefit in analyzing pulmonary fibrosis progression. Further validation is, however, needed for clinical translation. The combination of these biomarkers provided a more detailed and accurate picture compared to the isolated use of each. This multidisciplinary approach, which integrated clinical data with omics data, could enable more precise identification and monitoring of the disease and potentially pave the way for new treatment and management strategies. Furthermore, the use of machine learning techniques for data analysis further enhanced the ability to identify significant patterns and complex relationships between clinical and genetic variables.

Despite the promising findings, it is important to acknowledge that the implementation of these biomarkers—particularly when derived from exosomes isolated from bronchoalveolar lavage (BAL)—faces important technical and economic challenges in clinical practice. The isolation, characterization, and analysis of exosomal RNA currently require specialized equipment and expertise, which limits widespread routine use. Nevertheless, advances in microfluidics and RNA quantification technologies may gradually reduce these barriers, and alternative sources such as serum-derived exosomes are being increasingly explored to provide more accessible sampling options. Therefore, while BAL exosome profiling is not yet standardizable for routine diagnostics, our results suggest that this approach holds significant translational potential that warrants further investigation in larger, clinically oriented studies.

The Bayesian methodology used in this study represented a significant advance. Unlike pointwise generalized linear models (GLMs), which provide point estimates for coefficients, the Bayesian approach offers a posterior distribution for each coefficient. This not only provides a probability distribution as an output, but also allows for observing the influence of each variable on the classification.

In particular, the Bayesian approach allowed for an explicit evaluation of the uncertainty associated with parameter estimates, a crucial aspect in studies with small sample sizes. The posterior distribution of each coefficient provides a more accurate and robust estimate compared to traditional methods, such as generalized linear models (GLMs), which provide only point estimates. The probability distribution associated with each parameter allows for a more transparent assessment of each variable’s contribution to classification, with uncertainty indicated by the width of the distribution. This approach is especially useful in complex clinical contexts, such as in progressive pulmonary fibrosis, where classification models need to account for individual variability and a limited number of samples.

The distance of the coefficient from zero indicates the relevance of the variable, and the width of the distribution expresses the associated uncertainty. This approach improved the management of uncertainty in the data and provided more robust results, especially considering the limited sample size [58]. Furthermore, the ability to update prior probabilities with new evidence makes Bayesian analysis particularly suitable for exploratory studies in complex clinical contexts.

However, it is important to note that posterior uncertainty in many parameters remains significant. The width of the posterior distributions for several coefficients indicates substantial uncertainty in their estimates, which is reflected in the uncertainty observed in the classification results. This is particularly noticeable in models with more features, where the propagation of uncertainty from the coefficients increases the overall uncertainty in the classification. Thus, while the results are promising, the significant uncertainty suggests that a larger sample size would be beneficial to further refine the model and improve diagnostic and prognostic accuracy.

The small sample size led to significant uncertainty in the classification of diagnosis and prognosis. The Bayesian analysis enabled a quantification of this uncertainty, providing robust results but, at the same time, reporting high uncertainty in classifying some samples, thus suggesting that the model could be improved by including a larger number of patients. Furthermore, the lack of a control group of healthy subjects limits our ability to compare biomarker levels between different populations.

Beyond diagnostic and prognostic purposes, the identification of specific biomarkers such as KL-6, miR-21, and miR-92a, may influence future clinical management by enabling patient stratification and the tailoring of therapeutic approaches. For instance, miR-21 has been implicated in TGF-β-mediated fibrotic signaling pathways [54], making it a potential target for anti-fibrotic therapies. Recent preclinical studies have explored the use of miR-21 inhibitors to reduce fibrosis, suggesting that its measurement could guide the application of emerging RNA-based treatments [59,60]. Similarly, the modulation of miR-92a, known to affect endothelial function and cell proliferation [57], could open new avenues for therapeutic intervention in fibrotic diseases.

Moreover, the integrative use of biomarkers in predictive modeling may assist in identifying patients at higher risk of rapid progression, thereby informing decisions about the early initiation of anti-fibrotic agents, enrollment in clinical trials, or more intensive follow-up protocols. The Bayesian approach also offers the flexibility to incorporate new data over time, which is particularly relevant in evolving therapeutic landscapes where novel treatments—such as nintedanib or pirfenidone analogs, RNA therapeutics, or anti-miRNA agents—are under continuous development [61,62].

Overall, our findings highlight the potential of a biomarker-based, uncertainty-aware framework to support precision medicine strategies in progressive pulmonary fibrosis, by improving risk stratification, monitoring, and potentially guiding the use of targeted interventions.

In summary, our study highlights how the integration of KL-6 and miR-21 and miR-92a provides a more comprehensive view of progressive pulmonary fibrosis, improving both diagnosis and prognosis. The use of Bayesian methodology allowed for a more accurate assessment of uncertainty and influencing variables, facilitating the identification of new therapeutic strategies. This multidimensional approach marks a significant advancement in the management of pulmonary fibrosis, with the potential to optimize future treatments through larger studies and extended follow-ups.

## 5. Conclusions

This study demonstrates that Bayesian analysis can be a powerful tool for the diagnosis and prognosis of progressive pulmonary fibrosis (PPF), especially in contexts with limited data. The integration of genetic biomarkers such as miR-21, miR-92a, and KL-6 with traditional clinical parameters could enhance the management of patients with PPF, contributing to a more personalized approach. Specifically, miR-21 and miR-92a may be crucial for early diagnosis and monitoring disease progression, while KL-6 proves to be a useful prognostic indicator that could be incorporated into clinical routines to monitor patient response to antifibrotic treatments.

However, it is important to acknowledge that the integration of omics data into routine clinical practice presents several challenges. These include the need for larger validation studies, cost considerations, the availability of necessary technologies, and the integration of omics data with traditional clinical measures. Therefore, while these biomarkers hold promise, their clinical implementation requires careful consideration of these factors.

Further studies with larger samples and validation of biomarkers in independent cohorts are necessary to confirm these results. Additionally, exploring the use of exosomes derived from bronchoalveolar lavage and serum as diagnostic and prognostic tools could offer new opportunities to monitor local and systemic pathological processes, expanding our understanding of PPF and improving therapeutic strategies.

## Figures and Tables

**Table 1:** Basic statistics of the numerical features.

Feature	Min	Max	Mean	Std. Dev.	Missing
Age	46	80	66.46	7.56	0
FVC%	23	107	71.66	17.62	1
FEV1%	23	112	75.58	19.32	3
DLCO	30	122	57.51	19.08	4
DLCO/VA	50	167	100.67	26.05	9
Macro%	19	93	73.95	18.43	0
Neu%	0	74	12.71	16.16	1
Lin%	2	28	9.38	6.01	0
2^−ΔΔCT^ miR-21	0.07	3.41	0.81	0.95	0
2^−ΔΔCT^ miR-92a	0.02	3.31	0.5	0.64	0
2^−ΔΔCT^ KL-6	0.0	56.87	11.23	14.76	5

Abbreviations: FVC% = forced vital capacity (% predicted); FEV1% = forced expiratory volume in 1 s (% predicted); DLCO = diffusing capacity for carbon monoxide; DLCO/VA = DLCO corrected for alveolar volume; Macro% = percentage of macrophages in BAL; Neu% = percentage of neutrophils in BAL; Lin% = percentage of lymphocytes in BAL; 2^−ΔΔCT^ miR-21 = relative expression level of miR-21; 2^−ΔΔCT^ miR-92a = relative expression level of miR-92a; 2^−ΔΔCT^ KL-6 = relative expression level of KL-6.

**Table 2:** Feature grouping.

Functional Parameters	BAL Markers	miRNA Biomarkers
AGE	MACRO%	miR-21
FVC%	NEU%	miR-92a
FEV1%	LIN%	KL-6
DLCO		miR-21 × miR-92a
DLCO/VA		miR-92a × KL6

**Table 3:** Features considered for either diagnosis or prognosis.

Diagnosis	Prognosis
FVC%	Age
DLCO	DLCO
Lin	Lin
Neu	Neu
miR-21 × miR-92a	KL6
miR-21	miR-92a × KL6

**Table 4:** Selected features for either diagnosis or prognosis.

Diagnosis	Prognosis
miR-21 × miR-92a	KL6
miR-21	Age
Lin	DLCO

**Table 5:** Performance evaluation of the selected classification models for diagnosis (expected values, standard deviation in brackets; underlined: best values per column).

Model	Accuracy	Specificity	Sensitivity
Lin + miR-21 + miR-21 × miR-92a	0.75 (0.036)	0.67 (0.101)	0.81 (0.094)
Lin + miR-92a	0.70 (0.077)	0.58 (0.114)	0.81 (0.152)
Lin + miR-21 × miR-92a	0.72 (0.046)	0.62 (0.106)	0.80 (0.133)
Lin + miR-21	0.67 (0.050)	0.54 (0.118)	0.79 (0.143)
Lin	0.68 (0.046)	0.55 (0.141)	0.80 (0.168)
miR-92a	0.54 (0.048)	0.35 (0.307)	0.70 (0.328)
miR-21	0.52 (0.061)	0.34 (0.348)	0.67 (0.359)

**Table 6:** Performance evaluation of the selected classification models for prognosis (expected values) (expected values, standard deviation in brackets; underlined: best values per column).

Model	Accuracy	Specificity	Sensitivity
Age + KL6 + DLCO	0.75 (0.058)	0.73 (0.144)	0.77 (0.107)
DLCO + KL6	0.65 (0.052)	0.59 (0.184)	0.70 (0.168)
Age + DLCO	0.68 (0.057)	0.63 (0.171)	0.73 (0.135)
DLCO	0.62 (0.051)	0.51 (0.227)	0.71 (0.204)
Age + KL6	0.67 (0.056)	0.62 (0.155)	0.71 (0.138)
Age	0.58 (0.047)	0.51 (0.240)	0.64 (0.219)
KL6	0.57 (0.055)	0.55 (0.309)	0.59 (0.266)

## Data Availability

The data presented in this study are available on request from the corresponding author.

## Supplementary Materials

The following supporting information can be downloaded at: https://www.mdpi.com/article/10.3390/diagnostics15101257/s1, Supplementary Method S1. Exosome Purification from BAL; Supplementary Method S2. Western Blotting; Supplementary Method S3. Multiplex Surface Marker Analysis; Supplementary Method S4. RNA Extraction; Supplementary Method S5. Reverse Transcription and miRNA Expression Analysis by Real-Time PCR.
