# Supplementary material for: Bayesian Integration of Bronchoalveolar Lavage miRNAs and KL-6 in Progressive Pulmonary Fibrosis Diagnosis
Source: Diagnostics (Basel). 2025 May 15;15(10):1257. doi: 10.3390/diagnostics15101257 (PMC12109843; doi:10.3390/diagnostics15101257)
Supplement: Supplementary file 1 [file diagnostics-15-01257-s001.zip › diagnostics-3594066-supplementary.pdf]

Supplementary Materials

S1 Exosome purification from BAL

Exosomes were isolated from BAL samples by ultracentrifugation [58]. BAL samples were diluted with PBS, centrifuged for 30 minutes at 2000× g and then for 45 minutes at 12.000× g at 4°C. The obtained supernatants were filtered through a 0.22 μm filter and ultracentrifuged for two cycles of 70 minutes at 110.000× g to concentrate the exosomes in the pellet. Precipitated exosomes were resuspended in 200 μL of PBS and stored at -80°C until use.

S2 Western Blotting

The protein concentration of the exosomes was determined by Bradford analysis, as recommended by the manufacturer (BIORAD). 80 μg of proteins were subjected to electrophoretic migration in a 12% SDS-poly-acrylamide gel and transferred into PVDF membrane (BIORAD). The membrane was blocked with TBS/5% milk (Tris-buffered saline/0.1% Tween-20/5% low fat milk) and then incubated with the appropriate primary antibody diluted in blocking solution (CD81 (#10630D), Invitrogen; CD9 (#SC13118, Santa Cruz Biotechnology). After washings with TBS/0.1% Tween-20, the membrane was incubated for 1 hour with the secondary antibodies, diluted in the same buffer as previously described. Bands were visualized using ECL (BIORAD).

S3 Multiplex Surface Marker Analysis

MACSPlex analysis was performed using MACSPlex Exosome Kit - human(Miltenyi Biotec, Bergisch-Gladbach, Germany), capable of detecting 37 surface exosomal epitopes and two isotypic controls.

Samples containing exosomes were processed as follows: exosomes (4-20 μg of protein) were diluted with MACSPlex buffer (MPB) to a final volume of 120 μl and 15 μl of MACSPlex exosome capture beads were added. After an overnight incubation in the dark, at room temperature (RT), on an orbital shaker (450 rpm), 500 μl of MACSPlex Buffer were added to each tube; the tubes were then centrifuged at RT at 3.000 × g for 5 min. The supernatant was aspirated and 5 μl of MACSPlex exosome detection reagent CD9, CD63, and CD81 were added to each tube. The tubes were incubated, away from light sources, for 1 hour at RT in an orbital shaker (450 rpm). Subsequently, 500 μl of MACSPlex Buffer was added to each tube and incubated in the dark for 15 minutes at RT in an orbital shaker (450 rpm). Samples containing exosomes were centrifuged and the supernatant was aspirated, leaving about 150 μl in the tube. Flow cytometric analysis was performed at Facs Canto II followed by Kaluza Analysis 2.1 (Beckman and Coulter Life Sciences, CA, USA).

The concentrations of the epitopes present on the exosome surface were obtained from the ratio between [beads + exosomes + Ab] / [beads + Ab] of the corresponding controls.

S4 RNA extraction

Total RNA was extracted from BAL samples and exosomes using TRIzol reagent (Thermo Fisher Scientific, Waltham, MA, USA), according to the manufacturer's protocol. RNA concentration and quality were measured using the NanoDrop 1000 spectrophotometer (Thermo Fisher Scientific). RNA purity was assessed by the absorbance ratio at OD260/OD280.

S5 Exosomal miRNA and KL-6 Expression Profiling by qRT-PCR

Exosomal RNA was reverse transcribed into cDNA using the TaqMan MicroRNA RT Kit (Thermo Fisher Scientific), according to the manufacturer's instructions. The identified miRNAs were evaluated by real-time PCR using the TaqMan miRNA assay (Thermo Fisher Scientific), following the manufacturer's protocol.

The following mature miRNA sequences were used: hsa-miR-92a: UAUUGCACUUGUCCCGGCCUGU; hsa-miR-21: UAGCUUAUCAGACUGAUGUUGA.

RNU6B was used as an endogenous control [43]. Reactions were performed on the ABI-PRISM 7300 (PE Applied Biosystems). Concurrently, total RNA was reverse transcribed into cDNA using the iSCRIPT cDNA Synthesis Kit (Bio-Rad, Hercules, CA, USA), according to the manufacturer's instructions. The expression of the KL-6 gene was then assessed by qRT-PCR using SsoAdvanced™ SYBR® Green Supermix (Bio-Rad, Hercules, CA, USA), as specified by the manufacturer. Real-time reactions were set up in duplicate for each sample in 96-well plates with a reaction volume of 20 μL, containing 1X SsoAdvanced™ SYBR® Green Supermix, 250 nM of specific primers, and 100 ng of cDNA. β-actin was used as an endogenous control [44]. The primer sequences used for qRT-PCR amplification were as follows: - KL-6 – forward: 5′ AGACGTCAGCGTGAGTGATG 3′ – reverse 5′ GACAGCCAAGGCAATGAGAT 3′; - β-actin - forward: 5′ GACGACATGGAGAAAATCTG 3′ – reverse 5′ ATGATCTGGGTCATCTTCTC 3′. MiRNA's expression and KL-6 gene expression relative quantification was calculated using the comparative $2^{-\Delta\Delta Ct}$ method [45].
